# Distribution of Peripheral Memory T Follicular Helper Cells in Patients with Schistosomiasis Japonica

**DOI:** 10.1371/journal.pntd.0004015

**Published:** 2015-08-18

**Authors:** Xiaojun Chen, Wei Li, Yang Zhang, Xian Song, Lei Xu, Zhipeng Xu, Sha Zhou, Jifeng Zhu, Xin Jin, Feng Liu, Gengxin Chen, Chuan Su

**Affiliations:** 1 Department of Pathogen Biology & Immunology, Jiangsu Key Laboratory of Pathogen Biology, Nanjing Medical University, Nanjing, Jiangsu, P. R. China; 2 Chizhou Station for Schistosomiasis Prevention and Control, Chizhou, Anhui, P. R. China; Leiden University Medical Center, NETHERLANDS

## Abstract

**Background:**

Schistosomiasis is a helminthic disease that affects more than 200 million people. An effective vaccine would be a major step towards eliminating the disease. Studies suggest that T follicular helper (Tfh) cells provide help to B cells to generate the long-term humoral immunity, which would be a crucial component of successful vaccines. Thus, understanding the biological characteristics of Tfh cells in patients with schistosomiasis, which has never been explored, is essential for vaccine design.

**Methodology/Principal Findings:**

In this study, we investigated the biological characteristics of peripheral memory Tfh cells in schistosomiasis patients by flow cytometry. Our data showed that the frequencies of total and activated peripheral memory Tfh cells in patients were significantly increased during *Schistosoma japonicum* infection. Moreover, Tfh2 cells, which were reported to be a specific subpopulation to facilitate the generation of protective antibodies, were increased more greatly than other subpopulations of total peripheral memory Tfh cells in patients with schistosomiasis japonica. More importantly, our result showed significant correlations of the percentage of Tfh2 cells with both the frequency of plasma cells and the level of IgG antibody. In addition, our results showed that the percentage of T follicular regulatory (Tfr) cells was also increased in patients with schistosomiasis.

**Conclusions/Significance:**

Our report is the first characterization of peripheral memory Tfh cells in schistosomasis patients, which not only provides potential targets to improve immune response to vaccination, but also is important for the development of vaccination strategies to control schistosomiasis.

## Author Summary

Schistosomiasis affects more than 200 million people worldwide and causes more than 280,000 deaths per year. Current control strategies are based on chemotherapy, but recurrent reinfection of people living in endemic areas makes researchers search for an effective vaccine to provide long-term protection against schistosomiasis. The generation of long-lived high-affinity antibodies after vaccination is a pivotal step for anti-schistosome vaccine to eliminate schistosomiasis. Considering it is well-known that Tfh cells are specialized effector CD4^+^ T cells that provide help for germinal center (GC) formation and induce GC B cells to develop protective antibody responses, understanding the biology of Tfh cells in schistosomiasis patients is fundamental for vaccine strategy development. Here, for the first time, we documented increased frequencies of total and activated peripheral memory Tfh cells in schistosomiasis patients. Furthermore, we showed that Tfh2 cells were a major contributor to increased frequency of peripheral memory Tfh cells in patients with schistosomiasis japonica. More importantly, we found the significant correlations of the percentage of Tfh2 cells with both the frequency of plasma cells and the level of total IgG antibody in schistosomiasis patients.

## Introduction

Schistosomiasis remains a major public health problem in many developing countries. Estimates place the current number of infections at approximately 200 million people, with another 600 million considered at risk [1]. Although praziquantel remains highly effective in schistosomiasis treatment, it provides only short-term protection and does not block disease transmission or reinfection [2]. Furthermore, drug resistance and decreased susceptibility to praziquantel may occur with long-term use of the drug [3]. Thus, an effective vaccine against schistosome infection would be a major step towards eliminating this devastating and widespread tropical parasitic disease.

An effective anti-schistosome vaccine would immensely reduce the morbidity associated with schistosomiasis through induced immune responses leading to decrease in parasite load and reduced egg production [4,5]. The antibody dependent cell mediated cytotoxicity (ADCC) of effector immune cells such as eosinophils and macrophages has been suggested as one of the most important mechanisms of anti-schistosome vaccine-mediated protection [6–8]. Thus, the generation of long-term humoral immunity is a crucial component of successful vaccines. Interactions between T cells and B cells in germinal centers (GCs) are reported to be required for the generation of long-term humoral immunity [9]. Recent studies reveal that in GCs, a specialized subset of CD4^+^ T cells called T follicular helper (Tfh) cells, provide help to B cells to undergo proliferation, isotype switching and somatic hypermutation, resulting in long-lasting antibody (Ab) responses [10–12]. Thus, understanding the biological characteristics of Tfh cells in schistosomiasis patients is one of central issues to develop the vaccination strategies to control schistosomiasis.

In this study, we for the first time explored the characteristics of peripheral memory Tfh cells in patients with schistosomiasis japonica, which provides a better understanding of the role of Tfh cells in schistosomiasis and contributes to the development of the future vaccination strategies in schistosomiasis.

## Methods

### Ethics statement

Ethical clearance for this study was obtained from the Institutional Review Board of Nanjing Medical University, Nanjing, China (Permit Number: 2014NMUIEC001). The aims and objectives of the study were explained to each participant and written informed consent was obtained. All personal identifiers of the study notes and tapes were kept confidential and destroyed once the study was completed.

### Patients and healthy controls

The study was conducted on a total of 100 subjects, and all subjects were from a village in Chizhou City, Anhui province. The subjects included 50 healthy adult controls, 50 patients with schistosomiasis japonica by egg detection using the Kato-Katz method with duplicate examination of 3 consecutive stool specimens obtained from each individual [13]. The healthy controls did not display a history, laboratory or clinical signs of schistosomal infection, did not suffer from coinfections with HBV or HCV, and did not use medication two weeks before blood collection.

### Flow cytometry analysis

Human peripheral blood mononuclear cells (PBMCs) were collected into sodium heparin tubes (BD Biosciences, San Diego, CA) and purified by Ficoll-paque plus (GE healthcare, Sweden) density gradient centrifugation. Cells recovered from the gradient interface were washed twice, and stained for 30 min at 4°C with the following antibodies: CD3-FITC (clone HIT3a), CD4-Percp-Cy5.5 (clone RPA-T4), CD45RA-APC-H7 (clone HI100), CXCR5-Alexa Fluor 647 (clone RF8B2), PD-1-PE-Cy7 (clone EH12.1), ICOS-PE (clone DX29), CCR6-PE (clone 11A9), CXCR3-PE-Cy7 (clone 1C6), CD27-APC-H7 (clone M-T271), CD38-PE (clone HIT2), CD86-PE-Cy7 (clone 2331), CD19-APC (clone HIB19), all from BD Biosciences.

In brief, for total or activated peripheral memory Tfh surface marker analysis, cells were incubated with CD3-FITC, CD4-Percp-Cy5.5, CD45RA-APC-H7, CXCR5-Alexa Fluor 647, PD-1-PE-Cy7, ICOS-PE. For Th1, Th2, or Th17 surface marker analysis, cells were incubated with CD3-FITC, CD4-Percp-Cy5.5, CCR6-PE, and CXCR3-PE-Cy7. For Tfh1, Tfh2, or Tfh17 surface marker analysis, cells were incubated with CD3-FITC, CD4-Percp-Cy5.5, CD45RA-APC-H7, CXCR5-Alexa Fluor 647, CCR6-PE, and CXCR3-PE-Cy7. For circulating B cell surface marker analysis, cells were stained with CD3-FITC, CD4-Percp-Cy5.5, CD19-APC, CD27-APC-H7, CD38-PE, and CD86-PE-Cy7. The samples were fixed with 1% paraformaldehyde/PBS. Cells acquisition was performed using a FACSVerse cytometer (Lasers: 488 and 633; Mirrors: 507 LP, 560 LP, 665 LP, 752 LP, 660/10, and 752 LP; Filters: 488/15, 527/32, 568/42, 700/54, 783/56, 660/10 and 783/56, BD Biosciences). Data were analyzed with FlowJo (Tree Star, version 10.0.7).

To evaluate the percentages of GATA-3^+^ Tfh and Tfr cells, PBMCs were stained with CD3-FITC, CD4-Percp-Cy5.5, CD45RA-APC-H7, CXCR5-Alexa Fluor 647. Then, the cells were further intracellular stained with GATA-3-PE (clone L50-823, BD Biosciences) or FOXP3-PE (clone PCH101, eBioscience, San Diego, CA) after they were permeabilized with cold Fix/Perm Buffer (eBioscience). The samples were fixed with 1% paraformaldehyde/PBS. Cells acquisition was performed using a FACSVerse cytometer (BD Biosciences). Data were analyzed with FlowJo (Tree Star, version 10.0.7).

### Serology

To quantify the total serum IgG and IgE levels two commercial kit (Bethyl, Texas, USA) with established protocols from the manufacturer was used. Briefly, 96-well plates (Nunc MaxiSorp) were coated with 1 μg/well of capture antibody for IgE (catalog number A80-108A, Bethyl) or IgG (catalog number A80-104A, Bethyl) in Coating Buffer (0.05M carbonate-bicarbonate, pH 9.6) for 1 h at 25°C and blocked for 30 min with 200 μL/well Blocking Solution (50 mM Tris, 0.14 M NaCl, 1% BSA, pH 8.0). Between each step, the plates were washed 5 times with Wash Solution (50 mM Tris, 0.14 M NaCl, 0.05% Tween 20, pH 8.0). The serum from each patient was diluted 1:2 or 1:20,000 for IgE or IgG in Sample/Conjugate Diluent Solution (50 mM Tris, 0.14M NaCl, 0.05% Tween 20, 1% BSA), and 100 μL/well was added to the plates. Known concentration of purified human IgE (catalog number RC80-108-6, Bethyl) or IgG (catalog number RS10-110-4, Bethyl) was added to each plate to obtain a standard curve. Serum samples and standards were incubated for 1 h at 25°C. IgE or IgG bound to the plates was detected by the addition of HRP-anti-human IgE (catalog number A80-108P, Bethyl) or HRP-anti-human IgG (catalog number A80-104P, Bethyl) at 1:75,000 or 1:200,000 dilution in Sample/Conjugate Diluent Solution, followed by the addition of Substrate Solution (catalog number E102, Bethyl). After 15 min, the reaction was stopped with 100 μL of 0.18M sulfuric acid solution. Absorbance was measured at 450 nm using an automated ELISA reader (BioTek Synergy HT, Texas). For each patient, the amount of total IgE or IgG was quantified in triplicate.

### Statistical analyses

All data were analyzed using SPSS software (IBM, version 22). Significant Differences between specimens were determined by using Student’s *t* test or Mann-Whitney *U* test. Correlations were determined by Spearman’s ranking. The differences at p<0.05 were considered to be statistically significant.

## Results

### Increased frequencies of total and activated peripheral memory Tfh cells in patients with schistosomiasis japonica

**Fig 1:**
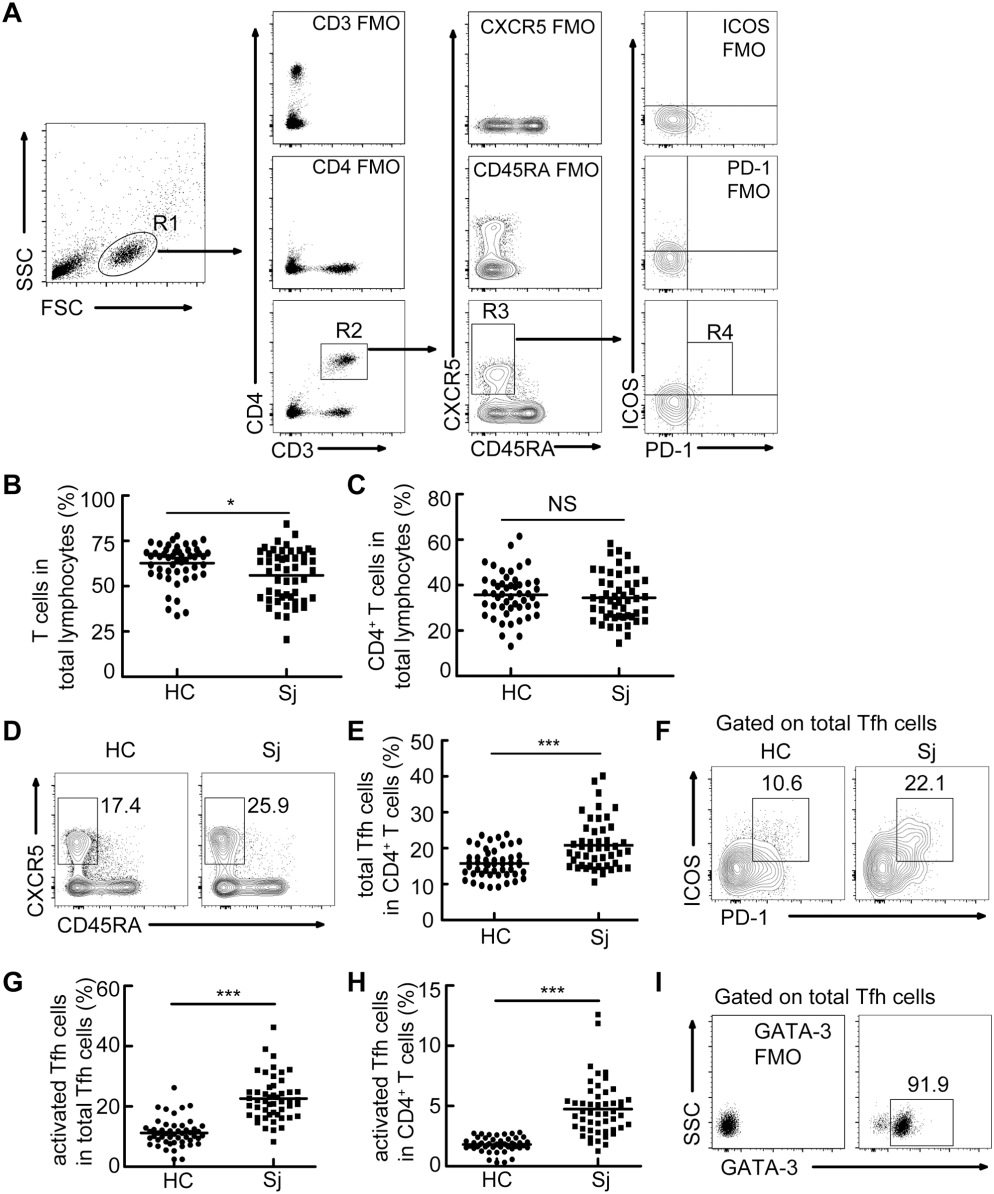
Increased frequencies of total and activated peripheral memory Tfh cells in patients with schistosomiasis japonica. PBMCs were isolated from healthy controls (HC, n = 50) and patients with schistosomiasis japonica (Sj, n = 50) and analyzed. (A) Gating schemes for analysis of the percentages of total peripheral memory Tfh (CD3^+^CD4^+^CD45RA^-^CXCR5^+^) and activated peripheral memory Tfh cells (PD-1^+^ICOS^+^ Tfh cells). PBMCs were stained with CD3, CD4, CD45RA, CXCR5, PD-1, and ICOS antibodies. Gating strategy defining total peripheral memory Tfh cells (R3), activated peripheral memory Tfh cells (R4); (B,C) Percentages of T cells (B) and CD4^+^ T cells (C) in total lymphocytes; (D-H) Representative flow cytometry data plots and statistics show the total peripheral memory Tfh cells (D, E), activated peripheral memory Tfh cells (F, G, H); (I) Representative flow cytometry profiles indicating the expression of GATA-3 in total Tfh cells. All flow cytometry results were analyzed and plotted using Fluorescence Minus One controls (FMO). *P<0.05, **P<0.01. ***P<0.001, NS indicating not significant.

Both our previous study [14] and other literature [15] described the increased frequency of Tfh cells in mice with schistosome infection. However, whether Tfh cells increase in percentage in schistosomiasis patients remains unknown. To study the biological characteristics of Tfh cells in patients with schistosomiasis, a total of 50 patients and 50 healthy controls were recruited. There was no statistically significant difference in the distribution of age or gender between patients and healthy controls (Table 1). The frequency of CD4^+^ T cells among total lymphocytes was comparable between patients and healthy controls, although the percentage of T cells was slightly lower in patients with schistosomiasis (Fig 1B and 1C). Next, we compared the frequencies of total peripheral memory Tfh cells (CXCR5^+^CD45RA^-^CD4^+^ T cells) [16] and activated peripheral memory Tfh cells (PD-1^+^ICOS^+^Tfh cells) [17,18] among CD4^+^ T or total peripheral memory Tfh cells in 50 patients with schistosomiasis japonica to 50 healthy controls. Results showed the increased frequencies of total and activated peripheral memory Tfh cells in patients with schistosomiasis (Fig 1A and 1D–1H). In addition, we found that almost all of the CXCR5^+^CD45RA^-^CD4^+^ T cells expressed high level of GATA-3 (Fig 1I).

**Table 1:** The demographic and clinical characteristics of subjects.

Parameters	HC	Schistosomiasis Japonica	P-value
Number	50	50	
Age(years)			
Mean±SD	58.5±8.2	61±9.1	>0.05
	(35–75)	(30–75)	(Mann-Whitney *U* test)
Sex N (%)			
Male	25 (50%)	30 (60%)	>0.05 (Pearson
Female	25 (50%)	20 (40%)	Chi-Square Test)

HC, healthy control

### Tfh2 cells and Tfr cells, but not Tfh1 or Tfh17 cells, were significantly increased in patients with schistosomiasis japonica

**Fig 2:**
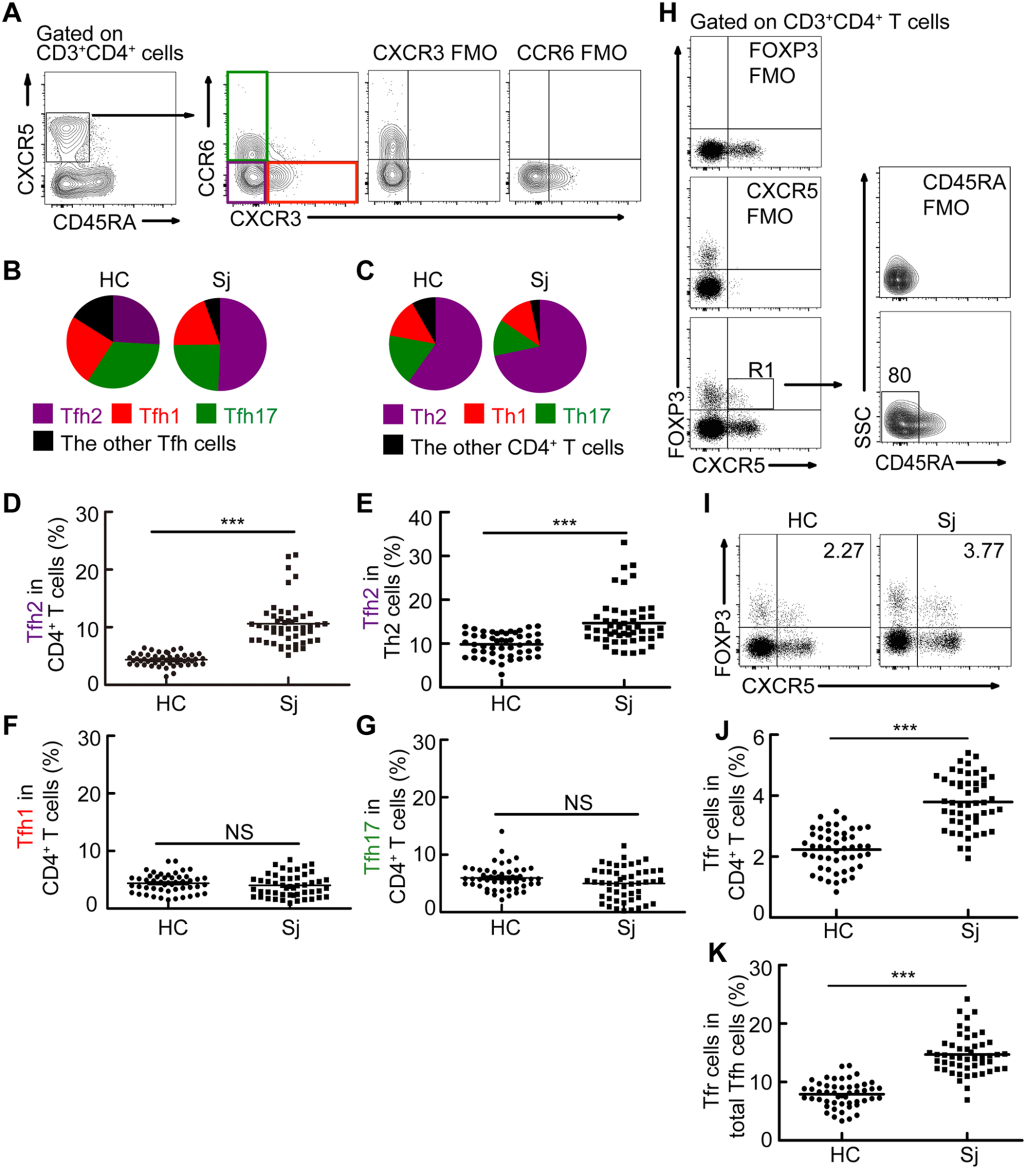
Tfh2 cells and Tfr cells, but not Tfh1 or Tfh17 cells, were significantly increased in patients with schistosomiasis japonica. (A) Gating schemes for analysis of the percentages of activated Tfh1, Tfh2 and Tfh17 cells. PBMCs were stained with CD3, CD4, CD45RA, CXCR5, CXCR3, and CCR6 antibodies. Gating strategy defining Tfh1 (CXCR3^+^CCR6^-^ Tfh cells), Tfh2 (CXCR3^-^CCR6^-^ Tfh cells), and Tfh17 cells (CXCR3^-^CCR6^+^ Tfh cells); (B) Shown is the distribution of Tfh1 (red), Tfh2 (purple), Tfh17 (green), and the other subsets (black) within total Tfh cells in healthy controls and patients with schistosomiasis japonica. Areas represent the means of percentages of Tfh1, Tfh2, Tfh17 or the other subsets with CR45RA^-^CXCR5^+^ cells. Gated to CR45RA^-^CXCR5^+^CD4^+^ T cells; (C) Shown is the distribution of total Th1 (CXCR3^+^CCR6^-^ CD4^+^ T cells, red), Th2 (CXCR3^-^CCR6^-^ CD4^+^ T cells, purple), Th17 (CXCR3^-^CCR6^+^ CD4^+^ T cells, green), and the other CD4^+^ T subsets (black) within total CD4^+^ T cells in patients with schistosomiasis japonica. Areas represent the means of percentages of Th1, Th2, Th17 or the other CD4^+^ T subsets with CD4^+^ T cells; (D-E) Frequencies of Tfh2 with CD4^+^ T cells (D) or total Th2 cells (E) in healthy controls or patients with schistosomiasis japonica; (F-G) Frequencies Tfh1 (F), and Tfh17 (G) cells within CD4^+^ T cells in healthy controls or patients with schistosomiasis japonica. (H) Gating schemes for analysis of the percentage of Tfr cells. PBMCs were stained with CD3, CD4, CD45RA, CXCR5, and FOXP3 antibodies. Gating strategy defining Tfr cells (R1); (I-K) Flow cytometry data plots and statistics show the Tfr cells within CD4^+^ T cells (I, J) or total Tfh cells (K) from one representative individual in indicated groups. All flow cytometry results were analyzed and plotted using Fluorescence Minus One controls (FMO). ***P<0.001, NS indicating not significant.

Evidence supports that peripheral memory Tfh cells in human can be subdivided into three major subsets with distinguished biological functions according to expression of CXCR3 and CCR6, Tfh1 (CXCR3^+^CCR6^-^CD45RA^-^CXCR5^+^CD3^+^CD4^+^ cells), Tfh2 (CXCR3^-^CCR6^-^CD45RA^-^CXCR5^+^CD3^+^CD4^+^ cells), and Tfh17 (CXCR3^-^CCR6^+^CD45RA^-^CXCR5^+^CD3^+^CD4^+^ cells) [19]. We then determined the distribution of peripheral memory Tfh-cell subsets in healthy controls and schistosomiasis patients. Results showed that Tfh2 cells were a predominant subset of peripheral memory Tfh cells, and accounted for more than 50% of total peripheral memory Tfh cells in patients with schistosomiasis (Fig 2A and 2B). In addition, the percentage of total Th2 cells, which include Tfh2 cells, was greater in schistosomiasis patients than those in healthy controls (Fig 2C). Furthermore, we found that the percentage of Tfh2 cells, rather than that of Tfh17 or Tfh1, is significantly increased in schistosomiasis patients (Fig 2D–2G).

Given that Tfr cells were identified as a Treg cell subset specialized for suppressing B and Tfh cells [20–22], we next investigated the biological characteristics of circulating Tfr cells in schistosomiasis patients. Results showed that most of circulating Tfr are CD45RA^-^ cells (Fig 2H), which is consistent with the previous observation that circulating Tfr cells have memory-like properties [22]. Furthermore, we found that the percentage of circulating Tfr cells within CD4^+^ T cells (Fig 2I and 2J) or total Tfh cells (Fig 2K) was significantly increased in schistosomiasis patients.

### Accumulation of Tfh2 cells was associated with increased frequency of plasma cells in patients with schistosomiasis japonica

**Fig 3:**
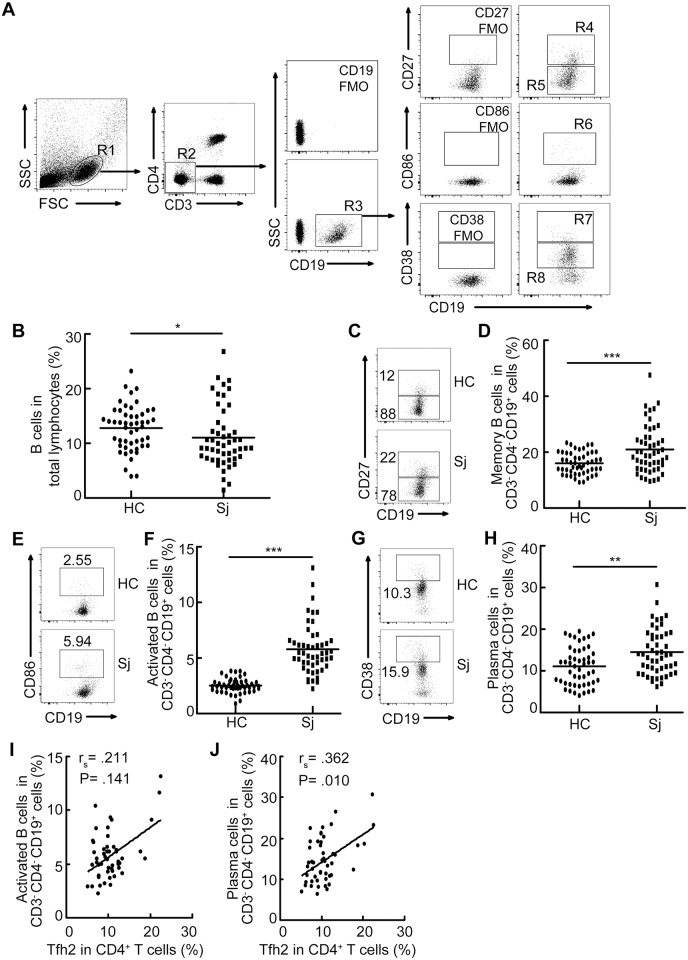
Accumulation of Tfh2 cells was associated with increased frequency of plasma cells in patients with schistosomiasis japonica. (A) Gating schemes for analysis of the percentages of different subsets of B cells. PBMCs were stained with CD3, CD4, CD19, CD27, CD86, and CD38 antibodies. Gating strategy defining memory B cells (R4), naive B cells (R5), activated B cells (R6), and plasma cells (R7); (B) Percentage of total B cells in total lymphocytes; (C-H) Representative flow cytometry data plots and statistics show the memory B cells (C, D), naïve B cells (C), activated B cells (E, F) and plasma cells (G, H); (I, J) The correlation analysis among the percentage of Tfh2 cells and activated B cells (I) as well as plasma cells (J). All flow cytometry results were analyzed and plotted using Fluorescence Minus One controls (FMO). **P<0.01. ***P<0.001.

Given the roles of Tfh cells in providing help to B cells, we next characterized the frequencies of different subsets of B cells by flow cytometry analysis. As shown in Fig 3, the percentages of CD27^+^CD19^+^CD3^-^CD4^-^ memory B cells [23–25], CD86^+^CD19^+^CD3^-^CD4^-^ activated B cells [26–28], and CD38^++^CD19^+^CD3^-^CD4^-^ plasma cells [29–31] in schistosomiasis patients were significantly greater than those in the HCs, although the percentage of total B cells was slightly decreased in patients with schistosomiasis. In contrast, the percentage of CD27^-^CD19^+^CD3^-^CD4^-^ naïve B cells [32] in patients was less than that in the HCs (Fig 3C). Furthermore, the percentage of Tfh2 cells was moderately correlated with the percentage of plasma cells (r_s_ = .362, p = .01) in schistosomiasis patients, and its correlation with the percentage of activated B cells (r_s_ = .211, p = .141) followed the same trend but it did not reach statistical significance (Fig 3I and 3J).

### Accumulation of Tfh2 cells was associated with increased level of IgG antibody in patients with schistosomiasis japonica

**Fig 4:**
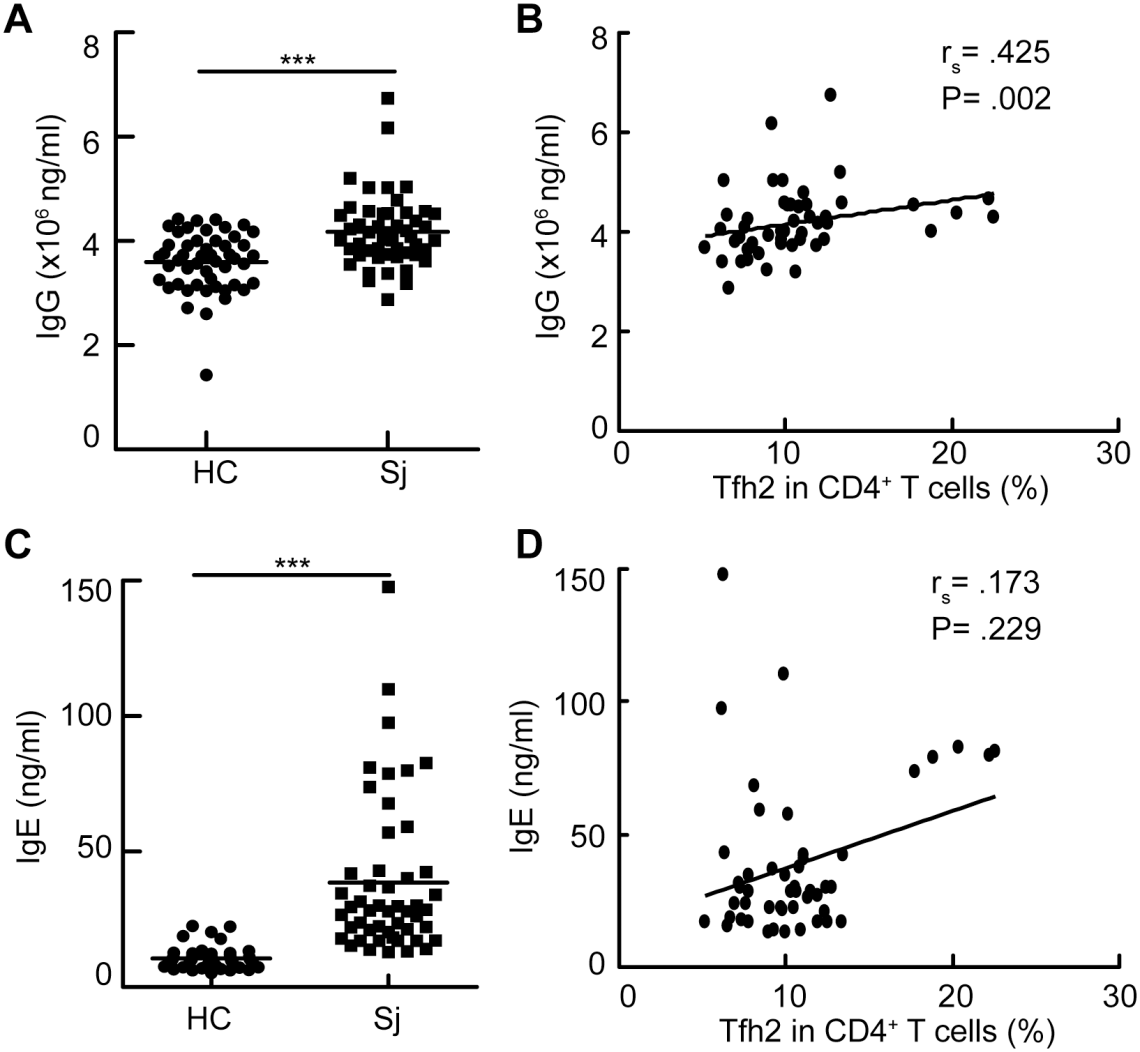
Accumulation of Tfh2 cells was associated with increased level of IgG antibody in schistosomiasis patients. (A, C) The concentration of total IgG (A) and IgE (C) antibodies in the serum from schistosomiasis patients or healthy controls; (B,D) The frequency of Tfh2 cells was correlated with the levels of serum IgG antibody (A) or IgE antibody (B) in schitosomiasis patients.

Given that Tfh2 cells are efficient at promoting IgG and IgE secretion [33], we next determined whether the frequency of Tfh2 cells was associated with the levels of total IgG and IgE antibodies in schistosomiasis patients. Results showed the increased concentrations of total IgG [HC vs. Sj: mean = 359.7, 95% confidence interval (95% CI) = 343.6–375.7 vs. mean = 417.9, 95% CI = 398.2–437.5; p<0.001] and IgE antibodies (HC vs. Sj: mean = 10.6, 95% CI = 9.5–11.7 vs. mean = 38.6, 95%CI = 30.5–46.7; p<0.001) in schistosomiasis patients (Fig 4A and 4C). Furthermore, a striking correlation between the percentage of Tfh2 cells and the level of total IgG (r_s_ = .425, p = .002) was observed in schistosomiasis patients (Fig 4B). Although there was a tendency of correlation between the percentage of Tfh2 and the level of total IgE (r_s_ = .173, p = .229) in schistosomiasis patients, it did not reach statistical significance (Fig 4D).

## Discussion

Prevention and control of schistosomiasis demands an effective vaccine. T follicular helper cells have a pivotal role in the generation of the long-term humoral immunity and are proved to be one of crucial contributors of successful vaccines. However, the lack of knowledge about Tfh cells in schistosomiasis patients limits the ability to develop successful anti-schistosome vaccinations. Here, we characterized the distribution of peripheral memory Tfh cells in patients with schistosomiasis japonica. Our study significantly extends our understanding of Tfh cells in patients with schistosomiasis, which is helpful for vaccine design for the prevention of schistosome infection.

Although the phenotypes of *Bona fide* Tfh cells in GCs are easy to be analyzed by flow cytometry, it is not only difficult to get lymph nodes from schistosomiasis patients only, but from humans in general. Fortunately, studies in humans showed that peripheral memory Tfh cells share functional properties with *bona fide* Tfh cells in secondary lymphoid organs [17,18,33–36], indicating the analysis of peripheral memory Tfh cells by flow cytometry is an alternative approach to study the biological characteristics of *bona fide* Tfh cells in human. Here, for the first time we revealed that the percentages of total and activated peripheral memory Tfh cells were significantly increased in schistosomiasis patients. These findings are in accordance with our previous observation that Tfh cells are substantially increased in schistosome-infected mice [14].

Human peripheral memory Tfh cells can be divided into three major subsets with distinguished functions according to the analysis of CXCR3 and CCR6 expression, including in CXCR3^+^CCR6^-^ Tfh1 cells, CXCR3^-^CCR6^-^ Tfh2 cells and CXCR3^-^CCR6^+^ Tfh17 cells [19]. Results from staphylococcal enterotoxin B *in vitro* coculture experiment suggested that human blood Tfh2 and Tfh17,but not Tfh1, cells can help naive B cells to produce immunoglobulins via producing interleukin-21 [33]. More specifically, it suggests that Tfh2 cells are considered to be efficient at promoting IgG and IgE secretion, whereas Tfh17 cells promote IgG and IgA secretion [33]. Our data showed that the significant increase of Tfh2 cells is a major contributor to the increased frequency of total peripheral memory Tfh cells in patients with schistosomiasis japonica. These findings nicely connect to our observation that the levels of memory B cells, activated B cells, plasma cells as well as total IgG and IgE responses were considerably increased in patients with schistosomiasis. More importantly, both IgG and IgE antibodies have been reported to be essential components of protective immunity, and involved in the ADCC of eosinophils and macrophages against schistosome larvae [37–39], which is considered as one of the most important means of anti-schistosome vaccine-mediated protection [6–8]. Thus, Tfh2 cells, a predominant subset of peripheral memory Tfh cells in schistosomiasis patients, might be considered as a potential target to improve IgG and IgE responses to vaccination. However, Tfh2 cells secrete Th2 cytokines, i.e., IL-4, IL-5, and IL-13 [33]. Given that the prolonged excessive production of Th2 cytokines contributes to the development of hepatic fibrosis and chronic morbidity in schistosomiasis [40] and the progression of Th2-mediated pathology in some diseases, such as asthma and other infectious diseases caused by extracellular parasites [41], it is very important for us to consider adverse effect of anti-schistosome vaccines on triggering Th2-mediated inflammation responses, particularly on liver pathology in patients with a history of infection with *schistosoma japonica*, when we manipulate Tfh2 cells to enhance IgG and IgE responses to vaccination. Strikingly, we found that the frequency of circulating Tfr cells, which play a crucial role in GC responses by limiting Tfh and GC B cell numbers as well as plasma cells differentiation [20], was significantly increased in schistosomiasis patients.

In summary, our study for the first time described the distribution of peripheral memory Tfh cells, circulating Tfr cells, and B cells in patients with schistosomiasis japonica, which provides us a better understanding of the biological characteristics of these cells in patients with schistosomiasis.
